# MedVidDeID: Protecting privacy in clinical encounter video recordings

**DOI:** 10.1016/j.jbi.2025.104901

**Published:** 2025-08-29

**Authors:** Sriharsha Mopidevi, Kuk Jin Jang, Basam Alasaly, Sydney Pugh, Jean Park, Ashley Batugo, Sy Hwang, Eric Eaton, Danielle Lee Mowery, Kevin B. Johnson

**Affiliations:** a Department of Biostatistics, Epidemiology, and Informatics, Perelman School of Medicine, University of Pennsylvania, 423 Guardian Dr, Philadelphia, 19104, PA, USA; b Department of Computer and Information Science, University of Pennsylvania, Levine Hall, 3330 Walnut St, Philadelphia, 19104, PA, USA; c Department of Computer Engineering, Hongik University, Seoul, 04066, Republic of Korea

**Keywords:** De-identification, Healthcare, Protected health information, Audio-video, Privacy

## Abstract

**Objective:**

The increasing use of audio-video (AV) data in healthcare has improved patient care, clinical training, and medical and ethnographic research. However, it has also introduced major challenges in preserving patient-provider privacy due to Protected Health Information (PHI) in such data. Traditional deidentification methods are inadequate for AV data, which can reveal identifiable information such as faces, voices, and environmental details. Our goal was to create a pipeline for de-identifying AV healthcare data that minimized the human effort required to guarantee successful de-identification.

**Methods:**

We combined open-source tools with novel methods and infrastructure into a six-stage pipeline: (1) transcript extraction using WhisperX, (2) transcript de-identification with an adapted PHIlter, (3) audio de-identification through scrubbing, (4) video de-identification using YOLOv11 for pose detection and blurring, (5) recombining de-identified audio and video, and (6) validation and correction via manual quality control (QC). We developed two de-identification strategies to support different tolerances for lossy video images. We evaluated this pipeline using 10 h of simulated clinical AV recordings, comprising nearly 1.1 million video frames and approximately 72,000 words.

**Results:**

In Precision Privacy Preservation (PPP) mode, MedVidDeId achieved a success rate of 50%, while in Greedy Privacy Preservation (GPP) mode, it achieved a 97.5% success rate. Compared to manual methods for a 15 min video segment, the pipeline reduced de-identification time by 26.7% in PPP and 64.2% in GPP modes.

**Conclusion:**

The MedVidDeID pipeline offers a viable, efficient hybrid solution for handling AV healthcare data and privacy preservation. Future work will focus on reducing upstream errors at each stage and minimizing the role of the human in the loop.

## 1. Introduction

The healthcare industry is rapidly evolving, driven by the widespread adoption of digital technologies that improve patient care, improve efficiency, and enable new forms of medical and biomedical research. Among these technologies, audio-video (AV) data play an important role in different aspects of health care, including telemedicine, where patients receive care remotely; clinical training [1], where medical professionals are trained using real-world scenarios; and patient monitoring, where continuous observation is essential for managing chronic diseases [2]. Furthermore, AV data have secondary uses in research, facilitating more comprehensive analyses and supporting the development of multimodal Artificial Intelligence (AI) models [3–5].

As healthcare providers increasingly capture AV data, the volume of generated, stored, and analyzed data is growing exponentially [6]. This surge presents a new opportunity for multimodal data sharing for hypothesis generation, hypothesis testing, and model fine-tuning. For example, AV data have already been shown to add value to educators [7,8], researchers [9–11], and policymakers [12–14]. With this opportunity, there is an inherent cost, particularly in securing this sensitive information and adhering to privacy regulations like the Health Insurance Portability and Accountability Act (HIPAA) [15] alongside established de-identification guidelines like those detailed in the NIST Interagency Report [16]. Healthcare data contains some of the most sensitive information, including detailed records of patients’ health conditions, treatments, and personal identifiers. The consequences of a data breach involving such information can be severe, impacting both patients and healthcare providers [17]. Patients may face risks such as identity theft, discrimination, or other harm, like medical fraud. At the same time, healthcare providers could encounter legal penalties, financial losses, and a loss of patient trust.

Most de-identification and anonymization tools in healthcare focus on textual data, such as dates and proper names in electronic health records [18] or identifiers in radiological images [19]. However, these methods do not cover AV data that reveal identifiers such as faces [20], voices [21], and environmental details that can be used to identify individuals [22]. While manual de-identification is possible, it is time-consuming and labor-intensive, making the massive volumes of AV data generated in modern healthcare settings impractical. Additionally, manual processes are prone to human error and inconsistencies, which can compromise patient privacy [23,24].

To address the lack of automated AV de-identification tools, we developed MedVidDeID, a modular pipeline to address the unique challenges of de-identifying AV healthcare data, treating the audio and video components separately, and applying tailored methods to remove Protected Health Information (PHI). We detail each pipeline stage, from transcript extraction and de-identification to audio scrubbing, video obfuscation, and validation. We used simulated clinical encounter videos to assess the overall performance of MedVidDeIDas well as to assess the performance of the pipeline relative to exclusively manual methods, or using a hybrid manual and automated strategy.

### Statement of Significance

**Table:** 

Problem	The increasing use of audio-video (AV) healthcare data introduces significant privacy risks that reveal faces, voices, and other contextual details.
What is already known	Existing de-identification efforts in the medical space focus mainly on textual data or images, leaving AV modalities less explored. While manual techniques exist, they are time-consuming, error-prone, and impractical for large-scale data.
What This Paper Adds	This paper presents a modular pipeline that de-identifies audio and video components using a novel data management pipeline that allows the integration of otherwise disparate open-source tools, significantly reducing de-identification effort time compared to manual methods and ensuring patient privacy.
Who Would Benefit from This Knowledge	Healthcare researchers and stakeholders involved in patient care, medical education, health services research, and engineering research.

## 2. Related works

There have been numerous advances in text, audio, and video privacy preservation. Research efforts have focused on developing methods to ensure compliance with regulatory frameworks such as HIPAA, which require that PHI be removed or obscured before data are shared or analyzed. This section reviews the relevant literature by modality, such as text, audio, and video, and then discusses integrated approaches. The review highlights gaps in existing approaches that motivate the development of MedVidDeID.

Successful video de-identification requires the creation of a transcript, followed by removing identifiers in text, audio (spoken and non-word sounds), and images. Each of these processes has advanced in the last decade. Below, we will summarize the current state of de-identification for each communication modality.

The creation of a transcript using automated speech-to-text technologies is a critical first step. One challenge with current speech-to-text software is that it is typically designed to support well-formed sentences as opposed to authentically transcribed conversational speech from a patient-healthcare provider visit. Conversational speech includes complete sentences in lay language (e.g., “I’ve had high blood pressure since, oh, I don’t know, guess since I was a teenager” becomes “The patient has a long history of hypertension”) but also semantically meaningful sentence fragments (e.g., “Uh huh” in the middle of a long point made by the healthcare provider) and overlapping speakers, all of which should be preserved to understand issues such as patient engagement. Tools such as Whisper, Google Speech-to-Text, Amazon Transcribe Medical, and Microsoft Azure Speech Services have been evaluated for conversational speech, with Whisper [25] as the open-source industry standard. While tools such as OtterAI have shown competitive performance in some evaluations [26], these proprietary and cloud-based platforms have real or perceived risks to data privacy, including potential data breaches, lack of encryption, and requirements for Business Associate Agreements under HIPAA.

The field of text-based de-identification has evolved from early rule-based systems [27] and machine learning classifiers [28,29] to more advanced deep learning approaches [30,31]. Recent approaches employ architectures such as pre-trained bidirectional transformers [32, 33] to identify and redact PHI in Electronic Health Records (EHRs), enabling large-scale anonymization and facilitating secure sharing of clinical text for research and model development.

Audio data consists of two communication modalities: spoken words and audible non-word sounds (e.g., laughter, keyboard typing, intentional silence). In addition, audio data contains unique voice characteristics that are unique to the speaker. Existing approaches to de-identify audio include voice masking (i.e., adding sounds over voices to obscure their pitch), pitch-shifting (i.e., altering the frequency distribution of a sound), and segment-level redaction (i.e., replacing a sound with silence or a different sound, such as a beep) [34,35]. While these techniques preserve linguistic content, balancing data quality and privacy remains challenging due to errors in automated speech recognition, temporal alignment, and audio quality variability [34]. Moreover, comprehensive pipelines that integrate textual and audio de-identification are still limited. An existing platform from Surgical Safety Technologies is specific for surgical applications, making it unsuitable for primary care [36]. Glendor [37] provides a similar system, but the performance on clinical conversation is unclear.

Video data consists of both audio, as described above, and sequences of frames. Video frames are inherently identifiable if an individual has images on the public internet or in other digital resources that link images to identities. Faces, distinctive physical attributes, and environmental cues can be used for re-identification. Current computer vision techniques use models for object detection and pose estimation to de-identify individuals frame-by-frame, either by blurring identifiable features or by generating new faces to replace the original [38–41]. However, these solutions often address only isolated tasks, such as faces or objects. The result is a fragmented approach that requires numerous iterations through a given video to remove all identifiable features.

Given the progress achieved in each data modality with existing open-source tools, it is surprising that no single pipeline has yet emerged to leverage these advances fully. Therefore, our work explores integrating and extending existing tools into a unified, end-to-end pipeline. By doing so, we aim to support clinical data containing PHI in AV formats, providing a scalable and holistic framework for privacy-preserving data sharing and analysis.

## 3. Methods

Our pipeline, MedVidDeID, uses a modular approach, treating audio and video components of the data separately and applying customized de-identification methods to each modality, ensuring that all PHI is effectively removed or obfuscated before recombining them. To leverage established open-source tools for functionality, we developed a Pipeline Configuration Manager that orchestrates processing parameters across all stages, and an Artifact Management System that stores intermediate outputs from each stage, enabling both debugging and research access to individual components.

### 3.1. Pipeline overview

**Fig. 1.**
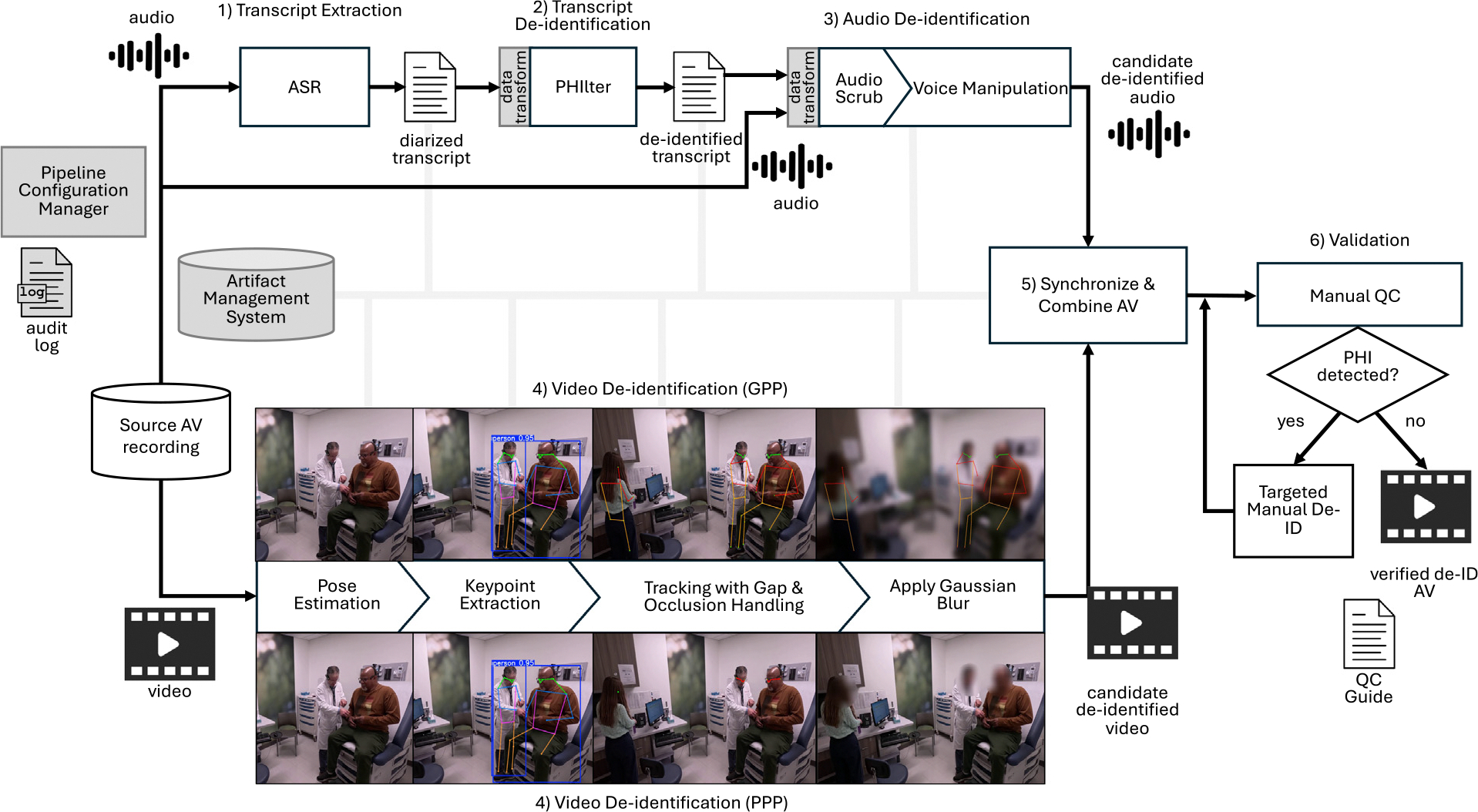
Diagram illustrating the different stages of the pipeline with two preservation modes. GPP: Greedy Privacy Preservation; PPP: Precision Privacy Preservation.

MedVidDeID, which can be accessed on Github (https://github.com/kbjohnson-penn/MedVidDeID), consists of six stages connected through custom integration components that transform, synchronize, and validate data as it moves through the pipeline. Unlike previous approaches that typically address only one modality (text, audio, or video), our pipeline conducts de-identification for all modalities. As shown in Fig. 1, our Pipeline Configuration Manager and Artifact Management System coordinate these stages, enabling reproducible processing workflows and maintaining the audit trails required for clinical research.

Fig. 1 summarizes the main stages and their interconnections. Each stage will be discussed below.

#### 3.1.1. Stage 1: Transcript extraction

In this stage, we utilize WhisperX [42], a state-of-the-art Automatic Speech Recognition (ASR) tool known for its accuracy and detailed output capabilities, such as generating sentence-level timestamps, word-level timestamps, and speaker diarization. The audio is extracted from the video files using an automated script and then processed through WhisperX. The high-accuracy large-v3 model is used to perform the transcription. Diarization settings are configured for patient-provider dialogues, typically involving two speakers. We also use Meta’s large pretrained and fine-tuned model Wav2Vec2-Large-960h to refine word-level alignment and retain overall synchronization between the transcript and the audio, ensuring that each recognized word is matched to its precise timestamp in the audio stream.

This stage outputs a JSON file containing the full transcript, including metadata such as speaker labels, timestamps, and confidence scores for each recognized word. This diarized transcript is stored in the Artifact Management System, enabling downstream processes and providing researchers direct access to the speech recognition output. This level of detail is necessary for the processes of audio deidentification, which relies on these timestamps to modify or obscure sensitive information.

#### 3.1.2. Stage 2: Transcript de-identification

Following transcript extraction, the next step in the pipeline is removing PHI from textual data. The transcript is de-identified using PHIlter [43,44]software, a robust natural language processing (NLP) tool designed to redact PHI under HIPAA Safe Harbor guidelines. PHIlter employs a rule-based system built from an extensive lexicon of medical terminology to identify and replace sensitive information, such as patient names, dates, and medical record numbers, with placeholders (e.g., “[NAME]”, “[DATE]”, “[LOCATION]”), thereby generating the transcript free of identifiable information. Our institution uses PHIlter’s ability to be locally customized to address specific requirements, including recognition of Penn Medicine-specific location names, provider names, and internal medical record number formats, expanded medical terminology dictionaries for specialized departments, and handling of institution-specific date formats and clinical note templates. We have also updated the module to accommodate varying inputs like plain text, Tab-separated values (TSV), JavaScript Object Notation (JSON), support customized inclusion and exclusion criteria, de-identify structured data in flat files, and improve configuration for the command line usage. These adaptations fit seamlessly into our workflows and enhance efficiency in handling diverse data needs.

Pre-processing of the Stage 1 transcripts is conducted in R version 4.2.2 by primarily leveraging the jsonline package and packages from the tidyverse. These tidyverse packages are used to transform and flatten the nested JSON-structured transcripts into a dataframe, then exported as a CSV file, the required input file format for deidentification in PHIlter. De-identification is conducted in a HIPAA-compliant limited performance computing cluster to increase processing efficiency and ensure the confidentiality required for handling PHI. For institutions without access to a secure computing infrastructure, a local implementation option is available in our GitHub repository, which includes basic security measures to de-identify a transcript.

Example of a sentence-level de-identified transcript snippet with timestamps:

50122 51163 So we’re back again.
51383 59630 So I’ve got a primary in **LOCATION**,
       and it’s time to get a primary here in **LOCATION**.
67358 68798 **NAME**, it’s nice to meet you.
69518 74239 I’m glad that you were able to find your way here,
       and hopefully we can help you going forward.


In the example above, named entities have been replaced with placeholders like “**LOCATION**” and “**NAME**” preserving context to the largest extent possible. Both the original and de-identified transcripts are versioned and stored in the Artifact Management System, maintaining a complete audit trail of all transformations.

#### 3.1.3. Stage 3: Audio de-identification

Once the transcript has been de-identified, with each placeholder timestamped, the next step is to ensure that the audio track is free from identifiable information. To achieve this, we developed AudioScrub, our custom audio de-identification module that performs two functions: (1) it parses PHIlter’s output to map each redacted entity to precise millisecond intervals in the audio stream, replacing these PHI segments with tones, and (2) it applies voice transformation to the remaining audio to prevent speaker re-identification.

For PHI removal, AudioScrub replaces identified segments with audio tones. We use a tone because silence may convey important information about patient-provider communication [45,46]. For voice transformation, AudioScrub uses WORLD[22,47], a high-quality speech analysis, manipulation, and synthesis system, to modify the fundamental frequency (F0) and scale the spectral envelope. This process randomly transforms the pitch and timbre, altering the speaker’s identity without changing speed, and doing so in a way that is difficult to reverse.

#### 3.1.4. Stage 4: Video de-identification

The primary approach for video de-identification involves detecting and obscuring facial features using state-of-the-art computer vision techniques. For this task, we used YOLOv11[48], a highly effective object detection and pose estimation model known for its accuracy and efficiency in identifying and tracking objects within video frames. YOLOv11 processes each video frame to identify individuals and their corresponding keypoints. We extract 17 keypoints for each person from these detections, including *𝑥* and *𝑦* coordinates and confidence scores. These keypoints, following the COCO pose estimation format, include 5 facial landmarks (nose, eyes, ears) and 12 body joints, allowing us to accurately identify the regions that need to be blurred, particularly the facial features.

MedVidDeID’s keypoint tracking algorithms then apply forward and backward linear interpolation to fill in any gaps caused by occlusions or rapid movements, ensuring continuous tracking of all keypoints. To further refine tracking, we use a Kalman filter on the keypoints, which smooths the trajectories and provides more accurate predictions of movements. The final obscuration method depends on the selected privacy mode. We implemented two distinct privacy preservation modes to accommodate different research needs:

Precision Privacy Preservation (PPP) Mode: A bounding box is calculated around facial keypoints, and a Gaussian blur is applied selectively to these regions, preserving environmental context.

Greedy Privacy Preservation (GPP) Mode: In addition to blurring faces, a Gaussian blur is applied to the entire frame. Keypoints are then overlaid on detected humans, effectively obscuring all environmental details while still preserving the general scene structure.

Both approaches maintain the video’s visual flow while achieving the desired level of privacy protection. The Pipeline Configuration Manager allows researchers to select the appropriate mode based on their research requirements, and all keypoint data and processing parameters are logged in the Artifact Management System.

#### 3.1.5. Stage 5: Combining audio and video

After separately de-identifying the audio and video components, our custom AV synchronization module, AVSync, aligns the processed streams by calculating the processing delay introduced in each stream, and using FFmpeg to multiplex the synchronized streams while preserving the original frame rate and audio sampling rate. AVSync ensures frame-level accuracy between the modified audio and video tracks, preventing AV drift. All processing parameters and synchronization offsets are logged in the Artifact Management System, along with the merged de-identified AV data.

#### 3.1.6. Stage 6: Validation

The final stage of the de-identification pipeline is the validation process, which is critical to ensure that de-identification has removed all PHI in the AV data. Validation begins with a manual Quality Control (QC) review, where the human reviewer follows a mode-specific QC guideline by listening to and watching the de-identified AV file. The validation criteria differ based on the privacy mode selected.

For PPP Mode validation, reviewers verify that: (1) the bounding box blur adequately obscures all facial features, (2) the blur region properly tracks faces during movement and occlusions, (3) environmental context remains visible and unaltered, and (4) no identifying features are visible outside the blurred regions.

For GPP Mode validation, reviewers confirm that: (1) the entire frame maintains consistent blur levels, (2) skeletal keypoints are clearly visible and accurately track human movement, (3) no environmental details or identifying information can be discerned through the blur, and (4) the keypoint overlay does not reveal facial features or other identifying characteristics.

In both modes, audio validation remains consistent, ensuring all PHI has been replaced with tones, and voice transformation prevents speaker identification. The reviewers follow specific guidelines to ensure consistency and thoroughness, particularly where the deidentification process may fail due to rapid movements, temporary occlusions, or complex interactions, such as those involved in physical examination.

If the reviewer identifies any remaining PHI or other identifiable information, the AV file is flagged as failing the QC process. In such cases, the AV file is manually de-identified, which may involve additional AV scrubbing, blurring, or adjusting blur based on the selected privacy mode. This further de-identification is completed by a second researcher, independent of the reviewer. All validation results, including QC pass/fail status, are recorded in the Artifact Management System.

**Fig. 2.**
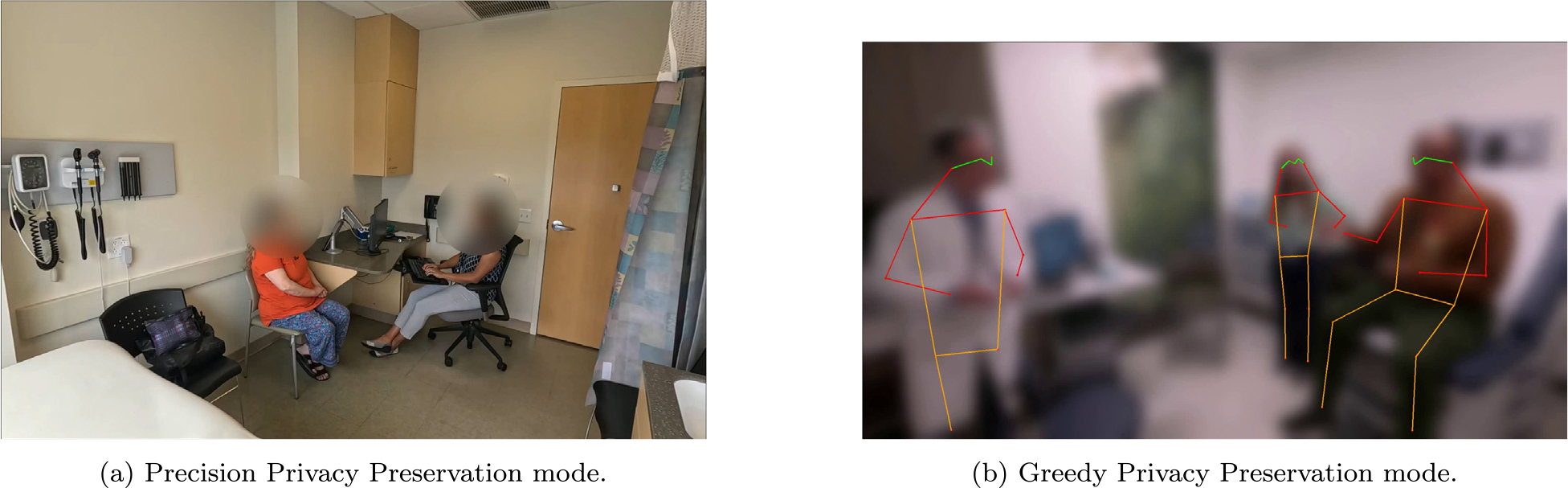
De-identified video frames.

After the AV file passes this validation process, it is considered fully de-identified and safe for sharing. Fig. 2 shows the output of both PPP and GPP modes after the final stage.

## 4. Results

The MedVidDeID pipeline was evaluated using a dataset comprising 9.9 h of simulated clinical AV recordings, totaling 591.7 min of patient-provider interactions. The dataset consisted of 1,064,046 video frames and 71,905 utterances. These recordings featured diverse genders, ages, and speech patterns. Recordings included typical clinical environment challenges such as background noise, interrupted visits, and overlapping speech. Additionally, PHI within the transcripts and audio was annotated to assess the effectiveness of PHI detection and removal. Tables 1 and 2 summarize the results of all the evaluations.

### 4.1. De-identification success

**Fig. 3.**
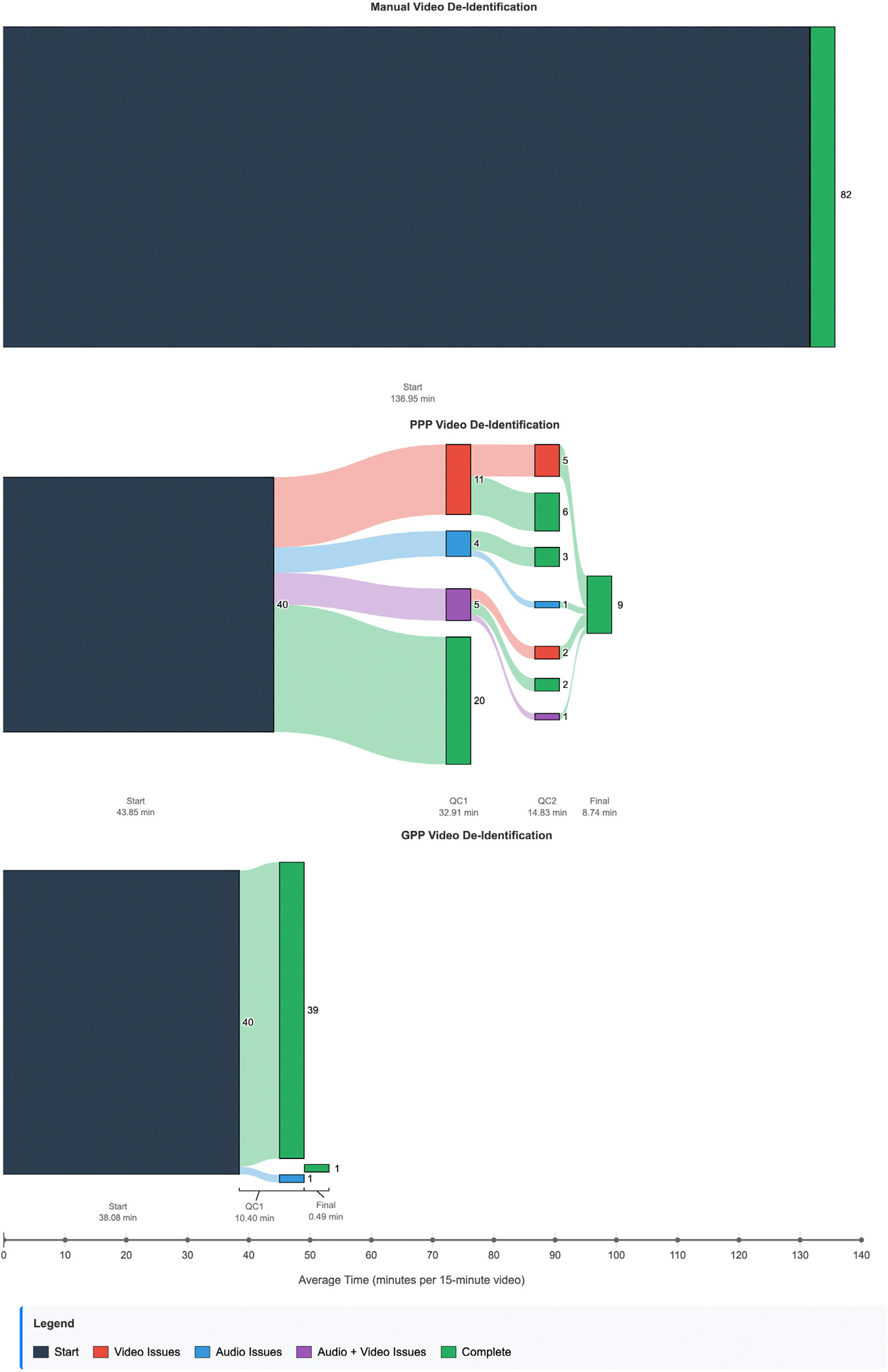
Sankey diagrams comparing the total time for manual editing vs. the MedVidDeID PPP vs. the MedVidDeID GPP.

Fig. 3 summarizes the overall success rate of the PPP and GPP strategies, where success was defined as no residual PHI in audio or video after a human review. In PPP mode, MedVidDeID achieved a success rate of 50%, while in GPP mode, it achieved a 97.5% success rate. AV recordings with residual PHI underwent a second round of QC and editing. Table 2 summarizes the PHI leakage and repair details for both PPP and GPP. In both GPP and PPP, audio PHI leakage was approximately one word for every 584 words processed. The efficiency of the pipeline was evaluated by comparing the processing times with those of manual de-identification methods. For a 15 min video segment, the manual processing time was 137 min, the PPP pipeline processing time was 100.3 min, the time reduction was 26.7%, the GPP pipeline processing time was 49 min, and the time reduction relative to manual processing was 64.2%.

* Of note, WhisperX was found to have an average WER (misrecognized or omitted words) of 18% not including any PHI.

The substantial reduction in processing time demonstrates the pipeline’s efficiency and potential to handle larger datasets without proportionally increasing the required time. This efficiency is particularly beneficial in research settings or clinical environments where timely data processing is essential.

### 4.2. Automated quality control

One approach to eliminating the need for a hybrid model involves using an adversarial approach to identify residual PHI. Therefore, we examined the effectiveness of using a second pass of our Video keypoint identification process using YOLOV8-face. We hypothesized that any remaining facial keypoints in a de-identified video would indicate frames that require additional attention. In a subset of 220,575 frames, the model’s precision and recall were around 3%. This severely limited the model’s usefulness as a screening tool, as most frames with faces went undetected, and the high false-positive rate created numerous false alarms that required manual review.

Fig. 3 summarizes MedVidDeID performance on a set of 40 videos, using both the Greedy Privacy Preservation and the Precision Privacy Preservation.

## 5. Discussion

Our modular audio-video (AV) de-identification pipeline, MedVidDeID, addresses an essential need in healthcare data security, especially with the increasing use of AV data in clinical settings and research. By integrating open-source tools and de-identification processes across audio and video modalities, our pipeline significantly decreases the effort and cost of creating HIPAA Safe Harbor-compliant AV datasets. This work extends prior research resulting from single-mode de-identification tools by integrating multimodal input into a single output artifact (the video) while also retaining the artifacts from each step in the pipeline.

According to our literature review, existing AV de-identification efforts focus on specialized applications, including anonymizing videos in the operating rooms [49] and audio-only de-identification frameworks [34], but no integrated tool or framework for general clinical encounters exists. MedVidDeID employs established tools in their respective domains, establishing performance baselines for end-to-end medical AV de-identification. As described in the text, we estimate that every 15 min of medical video processed by a hybrid pipeline using the GPP feature, with one person conducting a QC review and fixing what is found, results in a savings of 88 min per video. For 100 min of video, the GPP/hybrid model will result in 73 min of QC time, while the PPP/hybrid model will result in 376 min of QC time. We believe this savings is significant, both in terms of the absolute time required to pass individual videos, and in terms of the accuracy rate for the QC process, potentially eliminating the need for a second QC person to achieve higher levels of agreement.

The significant reduction in total de-identification time using MedVidDeID in any mode versus using a purely manual de-identification time suggests that MedVidDeID can meaningfully reduce the burden associated with human data manipulation [24,50]. This pipeline marks a step toward scalable and sustainable de-identification practices in research and clinical environments by minimizing the need for manual intervention.

Our results demonstrate a trade-off between precision and coverage in privacy preservation, as evidenced by the contrasting success rates of PPP and GPP modes. The choice between PPP and GPP may depend on the intended use of the de-identified AV data. For more ethnographic studies, GPP adds valuable information, such as positioning in the room, number of participants, etc. For projects using or developing computer vision, PPP may offer more details that enable understanding of how objects interact, body language, and attention. PPP may also improve trust in annotated video, especially if annotations are derived from analyzing the raw video. By providing both modes, we believe that MedVidDeID enables organizations to choose PPP when the need for richness outweighs the risk of minimal residual PHI, or when there are sufficient resources to perform and correct the results of quality control.

### 5.1. Challenges and limitations

Our pipeline contains a series of established tools that are used as developed, without alteration. Therefore, our performance is limited by errors intrinsic to the current version of these tools. For example, transcription accuracy was impacted by the Automatic Speech Recognition (ASR) model’s difficulty with overlapping speech, medical terms, and filler words. WhisperX, while effective, occasionally struggled with accurately transcribing sentences containing numbers such as “Vitamin B12”, missing interjections such as “okay” and “Right”, and filler words such as “um” and “uh”. Our reported 18.4% word error rate (Table 2) falls within typical ranges reported for medical conversation transcription, where error rates are between 9%–20% for various ASR systems in healthcare settings [26]. Domain-specific challenges such as medical terminology, overlapping speech, and informal dialogue commonly result in higher error rates than general speech recognition. We report overall transcription accuracy as our baseline metric. These omissions are particularly concerning for audio de-identification, as false negatives (i.e., missed words) could result in spoken PHI not being flagged for removal. This word error rate propagates through subsequent stages, occasionally affecting the accuracy of PHI detection and removal. Future work could additionally track PHI-specific word recognition rates to capture instances where PHI terms are mistranscribed rather than redacted. Measuring both general transcription accuracy and PHI-specific performance would provide a more complete assessment of audio de-identification systems. There is active research in ASR that we and others will evaluate and incorporate into this pipeline based on comparative analyses.

The realities of real-world conversation challenged named-entity recognition due to interjections, fillers, and informal language, which complicated the identification of PHI. Unlike medical documentation, spoken dialogues often include overlapping speech and colloquial expressions, making it difficult for PHIlter to detect and replace sensitive information. PHIlter’s 0.17% PHI leakage rate (Table 2) demonstrates the challenge of applying rule-based text de-identification to conversational transcripts, which differ substantially from the structured clinical notes these tools were originally designed for. Because PHIlter is a rule-based system that relies heavily on regular expressions, it operates primarily at a syntactic level, making it less effective with unfamiliar text types. To tackle these limitations, we could either expand the rule set or explore alternative NLP approaches, such as context-aware entity recognition models that are potentially better suited for conversational data, which could improve de-identification performance.

Voice transformations occasionally introduce noise artifacts that can disrupt listeners’ understanding. The transformation also made the voices sound robotic or childlike, which can diminish emotional cues that clinicians or researchers may want to analyze. There is active research on developing more refined voice transformation models that target multiple speakers without compromising emotional expressions, such as variational autoencoder-based voice conversion models [51] and voice conversion using deep embeddings [52].

Computer vision challenges included rapid movements and occlusions, computational intensity of the Gaussian blur process, and consistently identifying other distinguishing images beyond faces. Our combined YOLOv11 and custom tracking approach resulted in 78,104 frames requiring manual correction out of 1,064,046 total frames processed (7.3% frame error rate, Table 2). The frames requiring manual correction typically involved partial occlusions, reflections in glass surfaces or mirrors, and rapid transitions (e.g., lying down to sitting up, or starting to walk immediately) during physical examinations. Ensuring consistent de-identification beyond facial features, such as tattoos, ID cards containing names, and prosthetic devices, and automatically blurring information on computer screens presented additional challenges in detection and processing. Given these challenges and the critical importance of ensuring complete PHI removal in our initial repository of shareable data, we chose to implement GPP mode (complete frame blurring with keypoint overlay) as our default approach. This strategy prioritizes privacy protection while still preserving clinically relevant movement and pose information through the skeletal keypoints. As PPP mode accuracy improves and computational costs decrease, we will iteratively add more targeted video obfuscation techniques to the MedVidDeID pipeline.

Manual Quality Control (QC) and editing remain a major challenge. The process is time-consuming and inconsistent across reviewers due to subjective judgments about what constitutes adequate deidentification [23]. The development of a GPP and PPP mode was critical. It allows MedVidDeID to evolve as upstream unimodal deidentification tools improve. Undoubtedly, as improvements are made in the areas above, PPP’s success rate will increase, thereby reducing the time needed for human editing.

These component-level challenges reflect the current state of available tools when applied to healthcare’s unique requirements. Alternative approaches include transformer-based models for text de-identification [53], commercial ASR APIs for medical transcription [54], or specialized medical computer vision systems [55], each involving trade-offs in computational cost, customizability, or domain specificity. MedVidDeID successfully orchestrates the components into a complete, functional pipeline that achieves reliable de-identification through our hybrid approach. As underlying technologies improve, MedVidDeID’s modular architecture ensures it can incorporate advances while maintaining its core integration framework.

### 5.2. Future directions

Future work should focus on several key areas to address the challenges identified above. In particular, how can we improve the upstream ASR omission and commission errors? How do we improve the accuracy and completeness of PHI removal from text? And how can we automate our adversarial approach to systematically detect residual PHI once MedVidDeID has processed the data?

Among these challenges, automating quality control has an immediate opportunity for improvement. The manual QC process is currently a bottleneck, requiring extensive human review of entire videos. We recognize that there are improvements that could be implemented to reduce manual QC time. For example, our time estimate includes a complete review of a video, even after only a few seconds of PHI leakage is discovered, rather than employing a “punch list” of segments to re-review. In the future, we propose developing a targeted adversarial detection system that identifies the most common de-identification failures, exposed facial features, incompletely transformed voice segments that may reveal speaker identity, and visible PHI text, such as names or medical record numbers, detected through optical character recognition (OCR) and pattern matching. By implementing this automated screening, only flagged segments would require human review rather than complete manual QC, potentially reducing review time while maintaining rigorous safety standards.

Although the current version of MedVidDeID can be downloaded and run locally, we see opportunities to enhance its use in federated data collection settings. For instance, local execution enables sitespecific customization of tools like PHIlter, whose rule-based methods benefit from regional dictionaries to more accurately detect PHI. Similarly, artifact outputs and audit logs are stored locally, supporting privacy and control. These customizations are best handled at the site level; however, they shift the responsibility for validating named-entity removal and consolidating de-identified artifacts onto each participating site. To support multisite collaborations more effectively, future versions of MedVidDeID may require new components or refinements to existing open-source tools.

In addition, we recognize that this is only a starting point for AV de-identification in healthcare. Community-driven challenges such as the ImageNet Large-Scale Visual Recognition Challenge (ILSVRC) [56] and the Medical Image Computing and Computer Assisted Intervention Society (MICCAI) Grand Challenges have quickly driven advancements and visibility for novel techniques. We are exploring a similar challenge model to encourage collaboration and inspire new solutions to enhance MedVidDeID.

## 6. Conclusion

In this paper, we implemented MedVidDeID, a modular pipeline to address de-identifying AV healthcare data challenges. In Precision Privacy Preservation (PPP) mode, MedVidDeID achieved a success rate of 50%, while in Greedy Privacy Preservation (GPP) mode, it achieved a 97.5% success rate. Compared to manual methods for a 15 min video segment, the pipeline reduced de-identification time by 26.7% in PPP and 64.2% in GPP modes. The MedVidDeID pipeline offers a viable, efficient hybrid solution for handling AV healthcare data and privacy preservation.

## Figures and Tables

**Table 1:** Comparison of de-identification methods: Time efficiency and success rates.

Method	First-round success rate (%)	De-identification time (per 15 min of video)	Time reduction Rate (%)

Manual	100	137	(index)
PPP	50	100.3	26.7
GPP	97.5	49.0	64.2

**Table 2:** Summary of performance metrics for individual components of the pipeline.

Metric	Value

WhisperX ASR	
Word Error Rate (WER)	18.4%*
PHIlter Text De-identification	
PHI Leakage Rate	0.17%
YOLOv11 + Tracking Algorithm	
Frames with PHI leakage	7.3%
